# Comparison of brain normalization software and lesion compensation techniques in chronic perinatal stroke imaging

**DOI:** 10.1162/IMAG.a.1048

**Published:** 2025-12-03

**Authors:** Gillian N. Miller, Clara J. Steeby, Jorge Ortega-Márquez, Alberto Castro Palacin, Carrie Chui, Kenda Alhadid, Alyssa W. Sullivan, Aaron D. Boes, Patricia L. Musolino, Alexander L. Cohen

**Affiliations:** Department of Neurology, Boston Children’s Hospital, Harvard Medical School, Boston, MA, United States; Department of Neurology, Massachusetts General Hospital, Harvard Medical School, Boston, MA, United States; Department of Psychological and Brain Sciences, University of Iowa, Iowa City, IA, United States; Department of Psychiatry, Massachusetts General Hospital, Harvard Medical School, Boston, MA, United States; Departments of Pediatrics, Neurology, Psychiatry, Carver College of Medicine, University of Iowa, Iowa City, IA, United States; Iowa Neuroscience Institute, University of Iowa, Iowa City, IA, United States; Center for Brain Circuit Therapeutics, Brigham and Women’s Hospital, Harvard Medical School, Boston, MA, United States

**Keywords:** registration, neuroimaging, MRI, perinatal stroke, focal lesions, spatial normalization

## Abstract

Neuroimaging research depends on registration, the alignment of patients’ brains to a template or standard space, to enable accurate comparisons across individuals. Decades of work have advanced our ability to accurately register typical brains, but registering atypical brains, such as those with injury or highly distorted anatomy, remains a challenge. In particular, registration of perinatal stroke imaging is often complicated by delayed injury identification, which results in imaging obtained during the chronic stage where secondary structural impacts are evident. While analyses in native space can be valuable for subject-specific investigations, group-level studies require registration to a common template space, which enables between-subject comparisons of lesion locations and their network correlates. Although many registration algorithms exist, as do various compensation techniques for focal lesions, it is unclear how effective they are when applied to the highly distorted anatomy often present in this chronic perinatal stroke imaging. Here, we quantitatively and qualitatively compared the performance of three registration algorithms (FNIRT, ANTs, EasyReg) in registering eleven variably distorted brains with perinatal stroke to a standard template using their default lesion-compensation techniques. We also assessed the impact of “brain grafting”, that is, inserting a healthy tissue mask in place of the defined lesion area prior to registration. Our findings show that ANTs and EasyReg are significantly more accurate than FNIRT for chronic perinatal stroke imaging, although all three software packages have marked difficulty with large lesions. Notably, brain grafting significantly improved the lesion mask normalization performance of FNIRT. In light of these comparisons, the recently released EasyReg appears to be an appropriate starting point for registering cohorts with chronic perinatal strokes, but we still emphasize the necessity of consistent visual inspection of registered brains.

## 1 Introduction

Registration is the process of aligning images, such as three-dimensional brain images, so that consistent locations correspond to the same anatomic structures and functional regions. Registration is often used to align multiple images from the same person (e.g., from different timepoints or modalities) and to align images from different individuals (e.g., across a patient cohort or to compare results between research studies). Brain registration algorithms typically use features of both the source and target images to find the transformation matrix and warp field that minimize the differences between said images, applying various linear, non-linear, and diffeomorphic transform calculations.

Spatial normalization is a specific use-case of registration in which individual brains are registered to a standard template or atlas space, often the MNI152/ICBM152 template (Fonov et al., 2009). Registration to a standardized space is important for accurate within and between group comparisons and for communication of findings in scientific literature. While algorithms generally handle “healthy” brains well, accurate spatial normalization becomes difficult when applied to brains with abnormal features. Algorithms often rely on the concordance of features in the source and target images, and thus have difficulty when features are different or not present in both (Liu et al., 2021); as is the case with focal lesions, altered development, atrophy, or atypical tissue features. As such, many large-scale studies exclude images with similar features as part of their quality control (Alfaro-Almagro et al., 2018; Lu & Yan, 2023; Morfini et al., 2023; Teves et al., 2023). Some populations categorically have these features, for example patients with imaging obtained years after a stroke, heterogeneous brain injury involving both hemorrhagic and ischemic components, or some forms of infiltrative tumor.

Patient cohorts with focal lesions are a particular point of interest due to the use of methods like voxel-wise lesion symptom mapping (Bates et al., 2003; Rorden & Karnath, 2004) and lesion network mapping (Fox, 2018). These methods are unique in that they estimate pre-injury or typical brain functioning and connectivity to evaluate how disruptions contribute to new-onset behaviors and symptoms, rather than focusing on post-injury changes (Bates et al., 2003; Fox, 2018; Rorden & Karnath, 2004). For this reason, it is important to represent the lesion-locations within a common pre-injury brain framework (Fonov et al., 2011; Mazziotta et al., 2001). Lesion-based research methods often focus on acute ischemic stroke, leveraging imaging obtained within hours to days of injury. These images typically have intact structural data, making normalization a relatively straightforward process. However, in many cases, imaging may not be acquired until months or years after the initial injury, when significant alterations and downstream sequelae have altered overall brain appearance in a way that negatively impacts the performance of registration algorithms. As a result, some lesion-based research methods necessitate manually tracing the initial lesion location directly onto an atlas target. Manual delineation remains the accepted reference method when automated lesion filling or normalization fails, despite being labor-intensive and prone to variability (Bennett et al., 2023; Lo et al., 2022). While stroke is often recognized quickly in adults, significant delays in identification are common in perinatal strokes, which occur between 20 weeks of gestation and 28 days of life (Dunbar & Kirton, 2019). The perinatal period has one of the highest stroke risks across the lifespan, but recognizing the injury is challenging given the often non-specific or absent acute symptoms (Gacio et al., 2015). However, given the increased risk of stroke in this period of development, as well as differences in outcomes compared to pediatric and adult stroke (Kirton & Deveber, 2013), perinatal stroke research—and by extension accurate spatial normalization in this setting—is critical.

Lesion compensation techniques, such as cost-function masking and brain grafting, attempt to mitigate the registration challenges related to the presence of focal lesions. Cost-function masking, which excludes the lesioned area from registration calculations, outperforms unassisted registration in brains with simulated lesions (Brett et al., 2001) and real chronic adult strokes (Andersen et al., 2010). Brain grafting, also called enantiomorphic normalization and lesion filling, replaces the lesioned tissue with “healthy” tissue from the contralateral hemisphere or a template brain (Nachev et al., 2008; Radwan et al., 2021). More recently, machine-learning-based methods have been applied to the task of neuroimaging registration and lesion compensation with promising results. Implementation of deep learning networks typically involves supervised training (providing a “ground truth”) or unsupervised training of an algorithm or model to perform a specific task. In lieu of training an individualized, cohort-specific model, which is not always feasible, open-source pre-trained models have also recently become available (i.e., EasyReg (Hoffmann et al., 2022; Iglesias, 2023)). While machine-learning models can have trouble adjusting for unique features they were not trained on, researchers have had some success training models on brains with real tumors (Han et al., 2020) and simulated strokes (Andresen et al., 2022).

While there are many registration algorithms available, it is unclear how well they perform on real-world clinical data with distorted anatomy and variable lesions. Here, we compare three publicly available brain registration software packages (FNIRT (Andersson et al., 2007; Woolrich et al., 2009), ANTs (Avants et al., 2011), and EasyReg (Hoffmann et al., 2022; Iglesias, 2023)) and their lesion compensation techniques on a challenging real-world test case: spatial normalization of clinical scans from patients with chronic appearing perinatal strokes. Our objective is to systematically evaluate the accuracy and robustness of these algorithms in handling the complex brain anatomies often found in chronic-stage perinatal stroke imaging and provide insights into their practical utility for VLSM/LNM and similar methods.

## 2 Methods

### 2.1 Patients

**Fig. 1.**
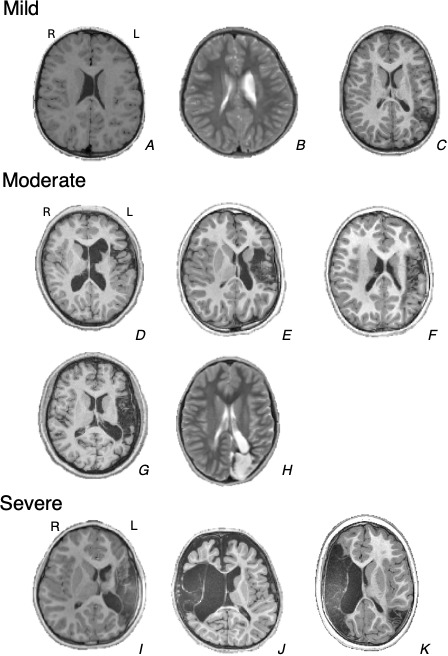
Representative slices from the 11 selected scans, classified as “mild”, “moderate”, or “severe” based on lesion volume.

Data for this study were selected from a large retrospective cohort of patients with perinatal strokes scanned clinically at Massachusetts General Hospital between 2005 and 2021. Data were obtained under protocols approved by the Institutional Review Board of Massachusetts General Hospital. Patient scans were considered for inclusion if lesions were clearly visible and collected at 2 years of age or later. From this subset, 11 scans were chosen to represent the range of overall brain deformation present in the cohort (Table 1, Fig. 1).

**Table 1.** Patient and lesion information.

Patient	Age (years)	Sex	Stroke type	Stroke laterality	Scan contrast	Image resolution	Lesion volume (%)	Lesion volume category
A	8.33	M	CVST, Hem	L	T1w	0.90 x 0.90 x 0.90	0.02	Mild
B	6.08	F	CVST, Hem	L	T2w	1 x 1 x 1	0.17	Mild
C	3.91	F	AIS	L	T1w	1 x 1 x 1	0.45	Mild
D	8.75	M	AIS	B	T1w	1 x 1 x 1	1.22	Moderate
E	7.5	F	AIS	L	T1w	0.90 x 0.94 x 0.94	1.43	Moderate
F	4.58	M	AIS, CVST	L	T1w	0.90 x 0.94.0.94	2.07	Moderate
G	15.25	M	AIS	L	T1w	1 x 1 x 1	2.70	Moderate
H	11.0	M	Hem	L	T2w	1 x 1 x 1	2.83	Moderate
I	6.5	F	AIS	L	T1w	1 x 1 x 1	5.97	Severe
J	4.25	M	AIS	R	T1w	1 x 1 x 1	7.52	Severe
K	4.08	M	AIS	B	T1w	0.90 x 0.94 x 0.94	12.27	Severe

M = male, F = female, AIS = arterial ischemic stroke, CVST = cerebral venous sinus thrombosis, Hem = hemorrhagic, L = left, R = right, B = bilateral.

We classified the 11 selected scans as either “mild” (lesion volume<1%; n = 3), “moderate” (lesion volume between 1 and 5%; n = 5), or “severe” (lesion volume>5%; n = 3). Lesion volume was calculated as the percentage of focal injury tissue out of the total brain volume at the time of the scan. As these were chronic scans, it should be noted that this may under- or over-represent the initial lesion volume; however, the relative anatomical relationships are typically intact, and the process of normalization partially re-approximates the initial volume. These scans were obtained between 3.91 and 15.25 years of age (mean age of 7.29 years) representing seven male and four female patients. The majority of included scans were left-sided strokes (n = 8), which are more commonly diagnosed within the perinatal stroke population (Fluss et al., 2019). Stroke types included arterial ischemic, cerebral venous sinus thrombosis, and hemorrhagic. All scans were obtained on 3.0T Siemens MRI scanners. For each patient, the best quality T1w structural image was selected for normalization; when not available, a T2w structural image was used (n = 2).

### 2.2 Data preprocessing and lesion definition

Selected scans were reoriented, bias-field corrected, and cropped with FSL-distributed anatomical processing tools prior to analysis (https://fsl.fmrib.ox.ac.uk/fsl/fslwiki/fsl_anat). Images were then skull stripped using the SynthStrip tool from FreeSurfer (Hoopes et al., 2022). Available T2w and FLAIR images were co-registered to the preprocessed T1w image for simultaneous viewing in ITK-SNAP (Yushkevich et al., 2016). If no T1w image was available, the T2w image was preprocessed as described above and used as the co-registration target for available FLAIR images. The above preprocessing steps were performed using our publicly available ‘bids-lesion-code’ pipeline (Cohen, 2020). Manual lesion segmentation was conducted following protocols and previously validated studies (Cariola et al., 2025; Everts et al., 2023; Lo et al., 2022; Vossough et al., 2024). First, an experienced lesion tracer (GNM) identified and delineated the lesions to create the lesion masks. These were then reviewed by a trained pediatric neurologist and neuroimaging specialist (ALC). Lesion segmentations aimed to capture the area of original insult, including areas of gliosis/glial scarring, encephalomalacia, and cystic encephalomalacia in the neighborhood of the initial injury.

### 2.3 Registration software

We selected three commonly used and publicly available software packages: FNIRT, ANTs, and EasyReg, to compare lesion compensation and brain normalization techniques in this perinatal stroke cohort. All registration packages were provided the same skull-stripped structural scans along with the corresponding 1 mm (T1w or T2w) MNI152 2009c nonlinear, asymmetric template target image (Fonov et al., 2009). An adult atlas, rather than a pediatric one, was selected as the registration target to test the algorithms’ ability to normalize the patient brains to the desired space directly. For ease of reproducibility, and consistency with common usage, each software was run with its respective default parameters. Resulting deformation fields were applied to the lesion masks using each software package’s corresponding application tool with nearest neighbor interpolation to maintain the binary nature of the lesion masks. Additionally, we repeated the above analysis with each software package after using “brain grafting” as an alternative lesion compensation strategy (described below).

#### 2.3.1 FLIRT/FNIRT

FNIRT (FMIRB’s Nonlinear Registration Tool; Andersson et al., 2007; Woolrich et al., 2009) is a well-established nonlinear registration tool from FSL that is used in conjunction with FMRIB’s Linear Image Registration Tool (FLIRT; Jenkinson et al., 2002). FLIRT is used to complete affine registration prior to running FNIRT. FNIRT operates purely on image intensity values, rather than modeling features such as brain structure and tissue type. As a result, FNIRT typically benefits when lesions, such as strokes and tumors, are “masked out” of the underlying cost-function optimization. This is done by providing a binary inverse lesion mask, where the lesion is labeled as ‘0’ and elsewhere is labeled as ‘1’, so that the lesioned area is excluded from registration calculations. The resulting warp field can then be applied to the patient-space lesion mask using FSL’s *applywarp* function.

#### 2.3.2 ANTs

ANTs (Advanced Normalization Tools; Avants et al., 2011) is an open-source toolkit for medical-image diffeomorphic registration and segmentation that integrates multiple linear, non-linear, and diffeomorphic registration and normalization steps via templated scripts. Here, we selected the commonly used *antsRegistrationSyN* script employing a three-stage transform: rigid, affine, then deformable symmetric normalization (SyN; B.; Avants et al., 2008). ANTs have been shown to work well for lesioned brains, even in the absence of an additional lesion compensation strategy (Ou et al., 2014). SyN with ANTs uses diffeomorphism to implement a large deformation framework, which is optimized via a cross-correlation measure of correspondence between the two images. Similar to FNIRT, ANTs operates on image intensity values, thus it can be beneficial to provide an inverse lesion mask to ANTs to implement cost-function masking in an attempt to compensate for a lesioned brain area. However, rather than completely omitting the lesioned area from the deformation field, ANTs employs constrained cost-function masking to estimate the deformation field for the lesion based on surrounding tissue, which has been demonstrated to improve performance (Kim et al., 2007). The resulting transform can then be applied to the lesion mask using the *antsApplyTransforms* function.

#### 2.3.3 EasyReg

**Fig. 2.**
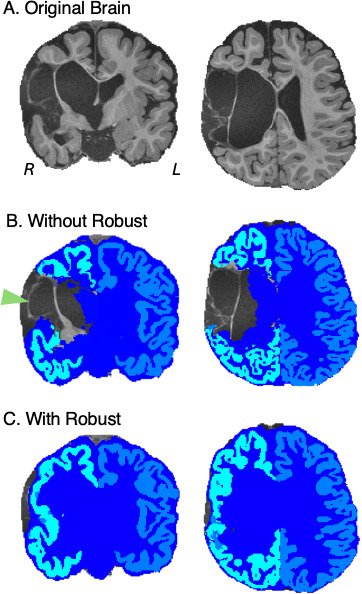
Representative brain segmentation created using SynthSeg v2.0 with and without the robust setting for a patient with a large cystic lesion (A). Note that without the robust setting (B), there is a lack of cortical ribbon delineation through the area of cystic lesion in the right hemisphere, while using the robust setting (C) generates a simulated cortical ribbon representation that - while not an accurate representation of the patient’s current anatomy - allows for boundary-registration algorithms to be utilized.

EasyReg is a recently released open-source, pre-trained deep learning-based registration tool (Hoopes et al., 2022; Iglesias, 2023) that is now distributed with the widely used FreeSurfer software package (Dale et al., 1999). It uses a trained convolutional neural network to compute deformation fields between two brain images, regardless of orientation or specific sequence (e.g., T1w, T2w, FLAIR, etc). EasyReg has no user-accessible parameters to tune and does not have specialized procedures for lesioned brains, though it was trained on brains with atrophy and white matter lesions in addition to “healthy” brains. As input, EasyReg requires a SynthSeg-produced (Billot, Greve et al., 2023) volume segmentation and surface parcellation for each target and source image. Here, we generated these segmentations prior to normalization using SynthSeg v2.0 with the “robust” setting and provided these segmentations to EasyReg. The robust flag has been shown to provide better performance in data with more variable intensity and resolution (Billot, Magdamo, et al., 2023), as is common in clinical data which are typically of “poorer” quality than research-standard protocols. Based on visual inspection, the robust setting, indeed, provided more liberal cortical representations in patients with large lesions (Fig. 2). After registering the structural images with EasyReg, the resulting warp fields were applied to the respective lesion masks using EasyWarp (Hoffmann et al., 2022; Iglesias, 2023).

### 2.4 Brain grafting

**Fig. 3.**
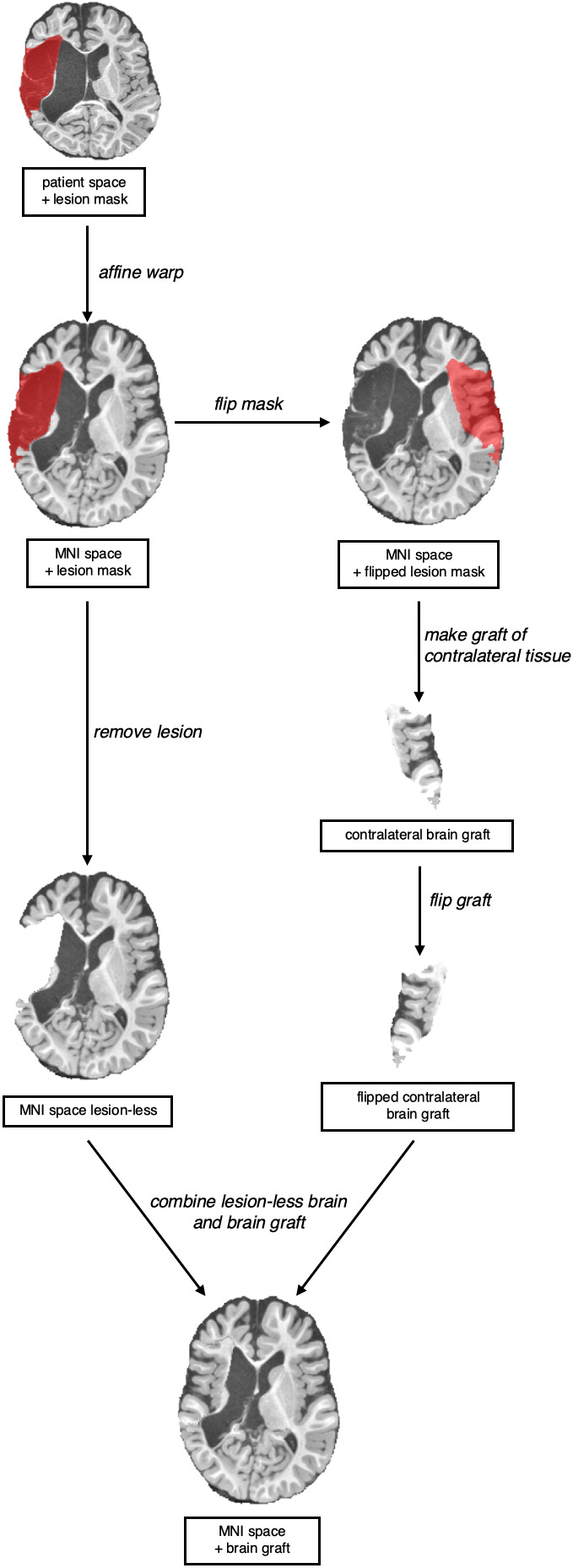
Brain grafting procedure. The patient’s structural image and associated lesion mask are affine warped to template space. The mask is then flipped onto the contralateral hemisphere and used to extract a “graft” of healthy tissue. In parallel, the lesioned area is removed from the structural image. The brain graft is then flipped back to the ipsilateral side and combined with the prepared structural image, replacing the area of lesioned tissue. This “corrected” image is then normalized by the three registration software packages.

Finally, we repeated the procedure described above after using “brain grafting” as an alternative lesion compensation strategy. Brain-grafted images were created for each patient’s skull-stripped images as follows (Fig. 3) (Nachev et al., 2008; Radwan et al., 2021). First, each patient’s brain and lesion mask were affine warped to the ICBM152 2009c asymmetric template (Fonov et al., 2009) using FLIRT - without cost function masking to improve hemispheric asymmetry, so homologous regions of the hemispheres were better aligned. Then, for patients with unilateral lesions (n = 9), a “brain graft” was created using the area in the “healthy” contralateral hemisphere that was homologous to the lesioned area. For patients with bilateral lesions (n = 2), where there is not a “healthy” contralateral hemisphere, brain grafts were created using the area in the ICBM152 2009c asymmetric template that was homologous to the lesioned area (Fonov et al., 2009, 2011; NIST / McGill University, 2009). No smoothing procedures were used. See Supplementary Figure 1 for example slices from all 11 patients.

The resulting brain-grafted images were then provided as the source image for each software package. We maintained the same default parameters and target image for fair comparison. As above, warps were applied to lesion masks using each tool’s built-in functions. In addition, the resulting warps were applied to the non-lesion filled brain using trilinear interpolation or, if not available, linear.

### 2.5 Evaluation measures

#### 2.5.1 Quantitative review

In order to assess the relative performance of the registration software packages in successfully recapitulating the lesion locations in the template brain, we compared the output of lesion segmentations hand-drawn in patient-space and then warped to the target template (i.e., “deformed labels”) to lesion segmentations that were hand-drawn directly onto the target template (i.e., “target labels”). In the latter case, the “registration” is done manually by the tracer, who attempts to recapitulate the size and spatial relationship of the lesion to surrounding intact structures, with the goal of denoting which voxels in the template brain are estimated to correspond to the initial injury. This includes maintaining the fractional distance to the ventricle and other landmark structures, capturing the fractional anterior to posterior extent of the injury relative to uninjured tissue. While not an ideal method for creation of target labels, hand drawing lesion segmentations directly on the target template is needed, in this case as it stands as the accepted method in the field. Both deformed and target lesion masks were drawn by an experienced lesion tracer (GNM) and then reviewed by a pediatric neurologist and neuroimaging specialist (ALC).

**Fig. 4.**
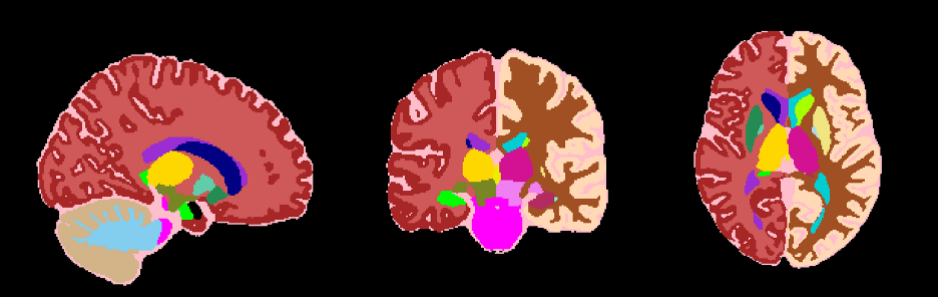
SynthSeg region labels: Cerebral White Matter*, Pallidum*, Lateral Ventricle*, 3rd Ventricle, Inferior Lateral Ventricle*, 4th Ventricle, Cerebellum White Matter*, Brain Stem, Cerebellum Cortex*, Hippocampus*, Thalamus*, Amygdala*, Caudate*, Accumbens Area*, and Putamen*, Ventral DC*. (* indicates regions with separate left and right labels.)

To analyze the normalization of the rest of the brain, region labels were created using FreeSurfer’s SynthSeg tool (Fig. 4) (Billot, Greve, et al., 2023). These regions were used as they are easily defined based on tissue appearance and contrast between regions and are less prone to interindividual differences of cortical folding or parcellation. We used all provided SynthSeg segmentation labels, except labels for cerebrospinal fluid and background, given that we used skull-stripped images. For each patient, “deformed labels” were created by segmenting the brain in patient-space and then transforming the labels to the target template using the calculated warp fields from each software package. SynthSeg’s coarse regional labels were chosen specifically because they are less sensitive to interindividual cortical folding patterns and therefore better suited for a consistent benchmark in structurally distorted brains. Voxels-of-region labels intersecting the lesion segmentation were excluded prior to applying the warp fields. For comparison, “target labels” were created by directly segmenting the target ICBM152 2009c asymmetric template. The template-space lesion masks described above were then used to exclude regions intersecting lesion areas for each patient.

Similar to previous work evaluating registration and normalization, the deformed and target labels were then used to calculate two volume-based similarity metrics: the Dice coefficient and Jaccard coefficient (Klein et al., 2009). The Jaccard coefficient is the intersection of a deformed label (S) and its analogous target label (T) divided by their total volume in number of voxels, accounting for voxels shared by both labels. This represents the degree of overlap between the label in the normalized brain and the target outcome.

$$J=\;\frac{\left|S\cap T\right|}{\left|S\cup T\right|}$$

The Dice coefficient is the intersection of a deformed label (S) and its analogous target label (T) divided by their total volume in number of voxels. This quantifies the similarity in volume of each label pair, allowing for comparison of the normalized brains to the target outcome.

$$D=2\frac{\left|S\cap T\right|}{\left(\left|S\;\right|+\left|T\;\right|\right)}$$

We conducted two standard fixed-effects three-way ANOVAs examining the main effect of lesion size and software, as well as their interaction effect, on normalization performance, as quantified by the Jaccard coefficient. We also included a main effect of the patient in this model to account for variance related to patient-specific factors. The first ANOVA aimed to assess the normalization of the lesioned area and therefore included the Jaccard coefficients calculated between the lesion deformed labels and lesion target labels for each patient and software (33 data points). This lesion data met all assumptions to allow for interpretable statistical results.

The second ANOVA aimed to assess the normalization of the brain outside the site of focal injury and therefore included the Jaccard coefficients calculated between the SynthSeg generated region deformed labels and region target labels for each patient and software (951 data points). These data did not meet assumptions for normality; therefore, we applied a Box-Cox transformation. After the transformation, the data still violated some assumptions of normality due to heteroscedasticity, however it now exhibited a clear single central tendency. This, combined with the large number of data points and the use of Generalized Least Squares models which are robust against heteroscedasticity, should allow this analysis to provide valid results due to the central limit theorem. Post hoc analyses for significant ANOVA results were performed using Tukey’s Honestly Significant Difference tests (Tukey, 1949).

In order to assess the impact of brain grafting on the normalization performance of each software, we created deformed and target lesion and region labels following the procedures described above for the patient brains normalized following the brain grafting procedure. Then, we calculated the Jaccard and Dice coefficients for these labels. In particular, we were interested in whether this would improve the performance of any algorithm compared to their default method, therefore we conducted three paired Wilcoxon Signed-Rank tests, one per algorithm, to determine if the label overlap was significantly different with and without brain grafting. A non-parametric test was chosen as the data were not normally distributed and contained outliers. We applied a Bonferroni correction for multiple comparisons and calculated Cohen’s d for significant results to assess effect size.

#### 2.5.2 Qualitative review

While the Dice and Jaccard coefficients help us to compare the normalization outcomes, they may not fully capture the nuances of each software’s performance. To better understand this and the specific changes resulting from each software, we also conducted a systematic qualitative visual review. A pediatric neurologist and neuroimaging specialist (ALC) visually reviewed all normalized brains and lesion masks for each patient in a blinded fashion and subjectively ordered them from “best” to “worst”. Independently, normalizations created with and without brain grafting were also visually compared to understand the impact of brain grafting on pairwise spatial normalization performance.

Within each patient, the normalized brains from each of the three software packages were visually compared and ranked from best to worst based on their recapitulation of the focal-injury area and the similarity of the rest of the brain to the target brain. Priority was given to reproducing the presumed location and extent of the original lesion. Penalty was given when surrounding structures were pulled into the lesion area, as well as for “over-warping” such as further enlarging ventricles, “pulling” the midline to one side, or misalignment of the brain edge. The comparative rankings were performed by focusing on the lesion location and extent in the original patient-space image, comparing this to the outputs of the algorithms. The ranking also considered the ability to compensate for enlarged ventricles, atrophy of subcortical structures, and asymmetry between the hemispheres, which impact the lesion mask’s relative location to surrounding brain structures.

## 3 Results

### 3.1 Quantitative results

As expected, the Jaccard and Dice coefficients were highly correlated (r = 0.995 and r = 0.984 for the lesion and region labels respectively). For clarity, analyses are reported with only the Jaccard coefficient, as results did not significantly vary between the two measures.

**Fig. 5.**
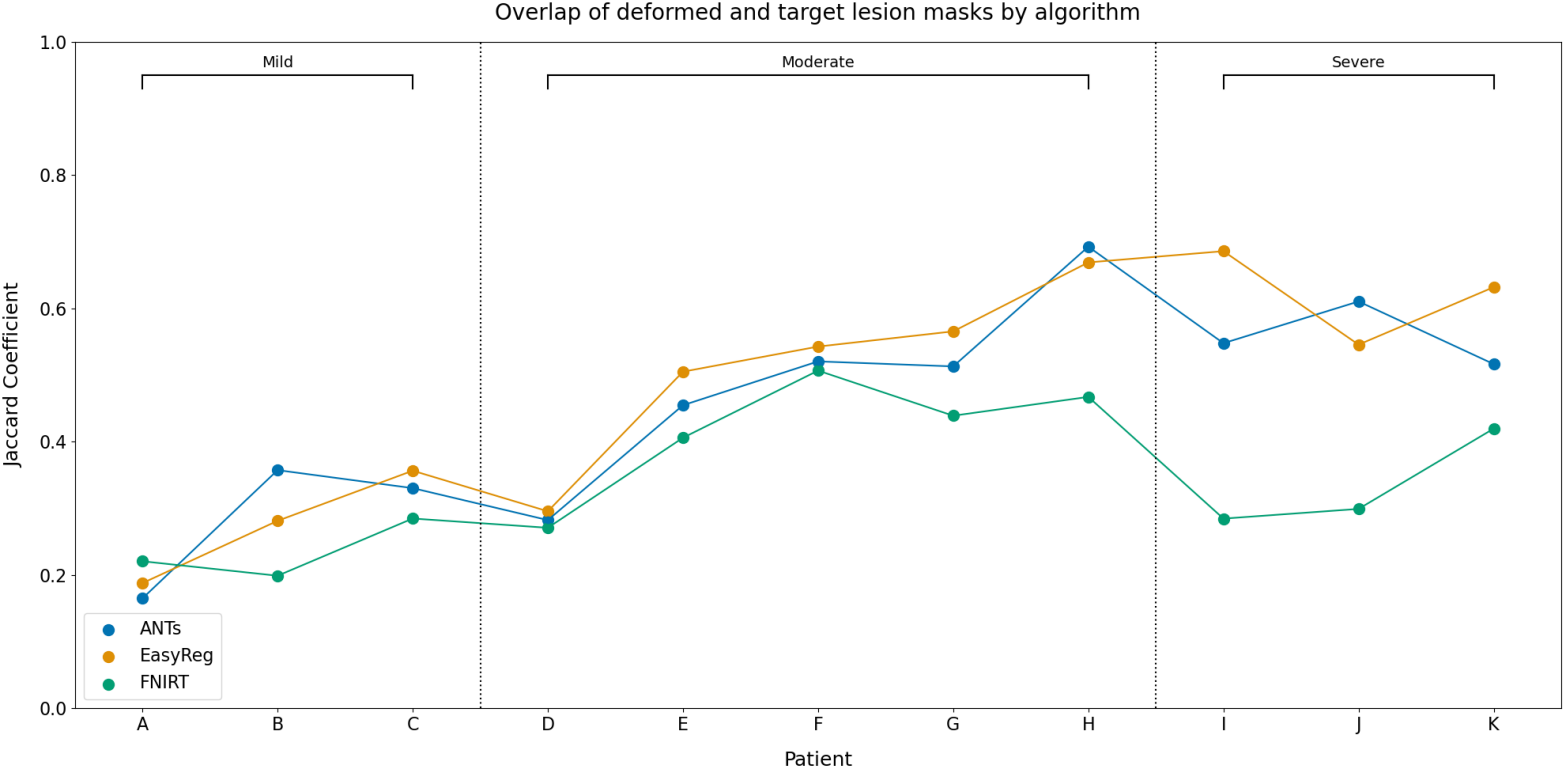
Jaccard coefficients representing the similarity between each deformed lesion mask and hand-drawn target mask for each patient and algorithm (default method), grouped by lesion volume classification.

We found a significant main effect of software (F(2, 16)=12.675, p = 0.0005) and a significant main effect of lesion size (F(2,16) = 40.678, p < 0.00001) on the Jaccard coefficients representing the degree of overlap of lesion deformed and target labels (Fig. 5). There was no significant interaction effect between software and lesion size on the degree of overlap of the deformed and target lesion masks (F(4, 16) = 2.293, p = 0.104). Tukey Honestly Significant Difference testing post-hoc tests revealed that these differences were driven by FNIRT’s reduced accuracy with the largest lesions compared to ANTs and EasyReg.

**Fig. 6.**
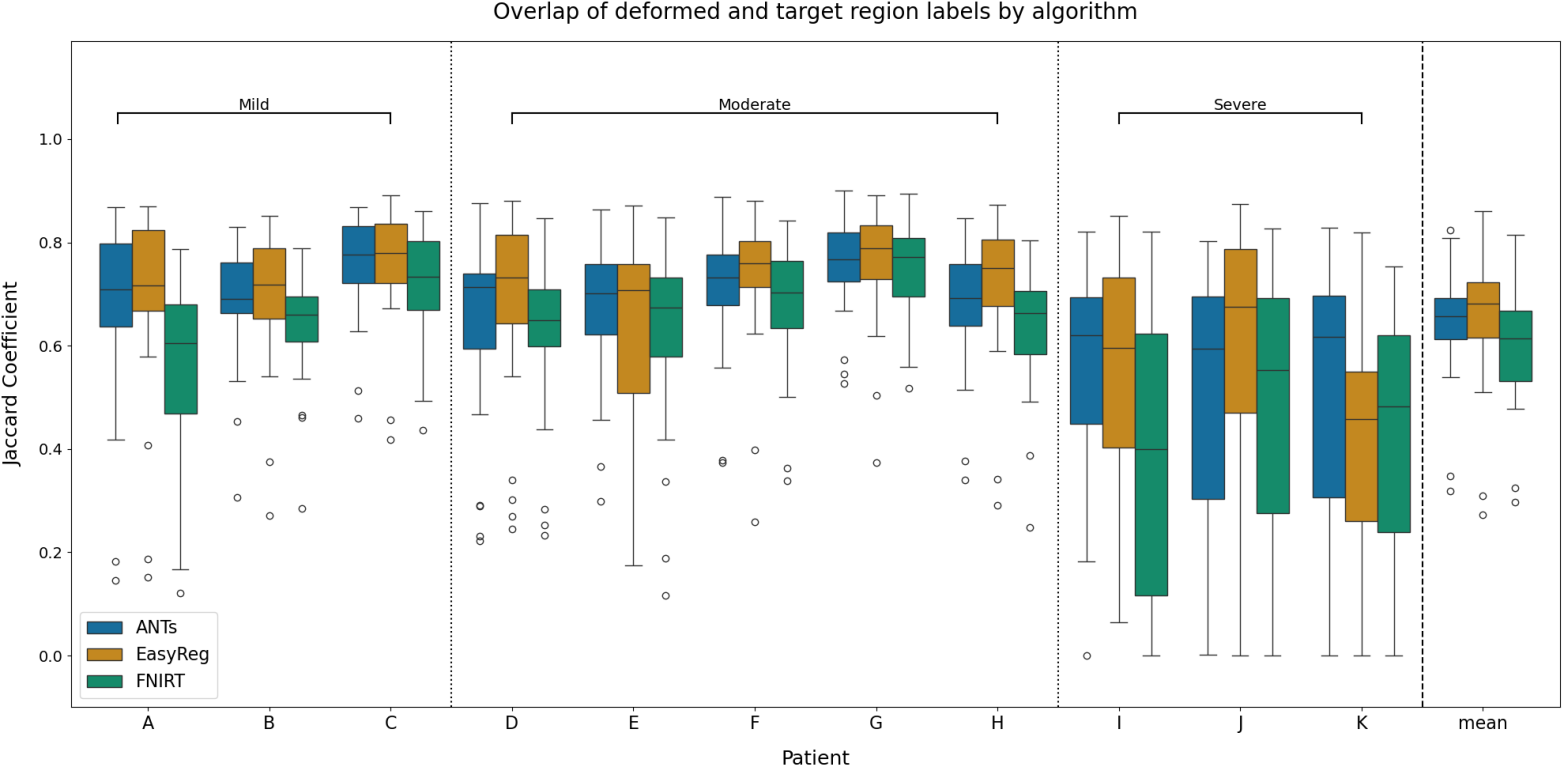
Distribution of Jaccard coefficients representing the similarity of deformed and target region labels for each patient and algorithm (default method), grouped by lesion volume classification.

Regarding the normalization of the rest of the brain, we found both a significant main effect of software (F(2, 934) = 19.260, p < 0.00001) and a significant main effect of lesion size (F(2,934) = 16.070, p < 0.00001) on the Jaccard coefficients representing the overlap of deformed and target labels (Fig. 6). There was no significant interaction effect between software and lesion size on the degree of overlap of the deformed and target labels (F(4, 934) = 8.152, p = 0.305). Post-hoc analysis via Tukey Honestly Significant Difference testing indicated that FNIRT performed significantly worse than ANTs (mean difference = -0.4598, p = 0.0003) and EasyReg (mean difference = -0.6156, p < 0.0001); ANTs and EasyReg did not significantly differ (mean difference = 0.1558, p = 0.4305). For the effect of lesion size, Tukey Honestly Significant Difference testing revealed that, overall, brains with larger “severe” lesions had significantly worse normalization than those with “mild” (mean difference = -1.0742, p = 0.1524) and “moderate” (mean difference = -1.2847, p < 0.001) lesions; the normalization of brains with “mild” and “moderate” lesions did not differ significantly (mean difference = 0.2105, p = 0.1524). Mean differences from post-hoc tests involving region labels refer to the Box-Cox transformed Jaccard coefficients and thus may exceed the native 0–1 range of the original metric.

**Fig. 7.**
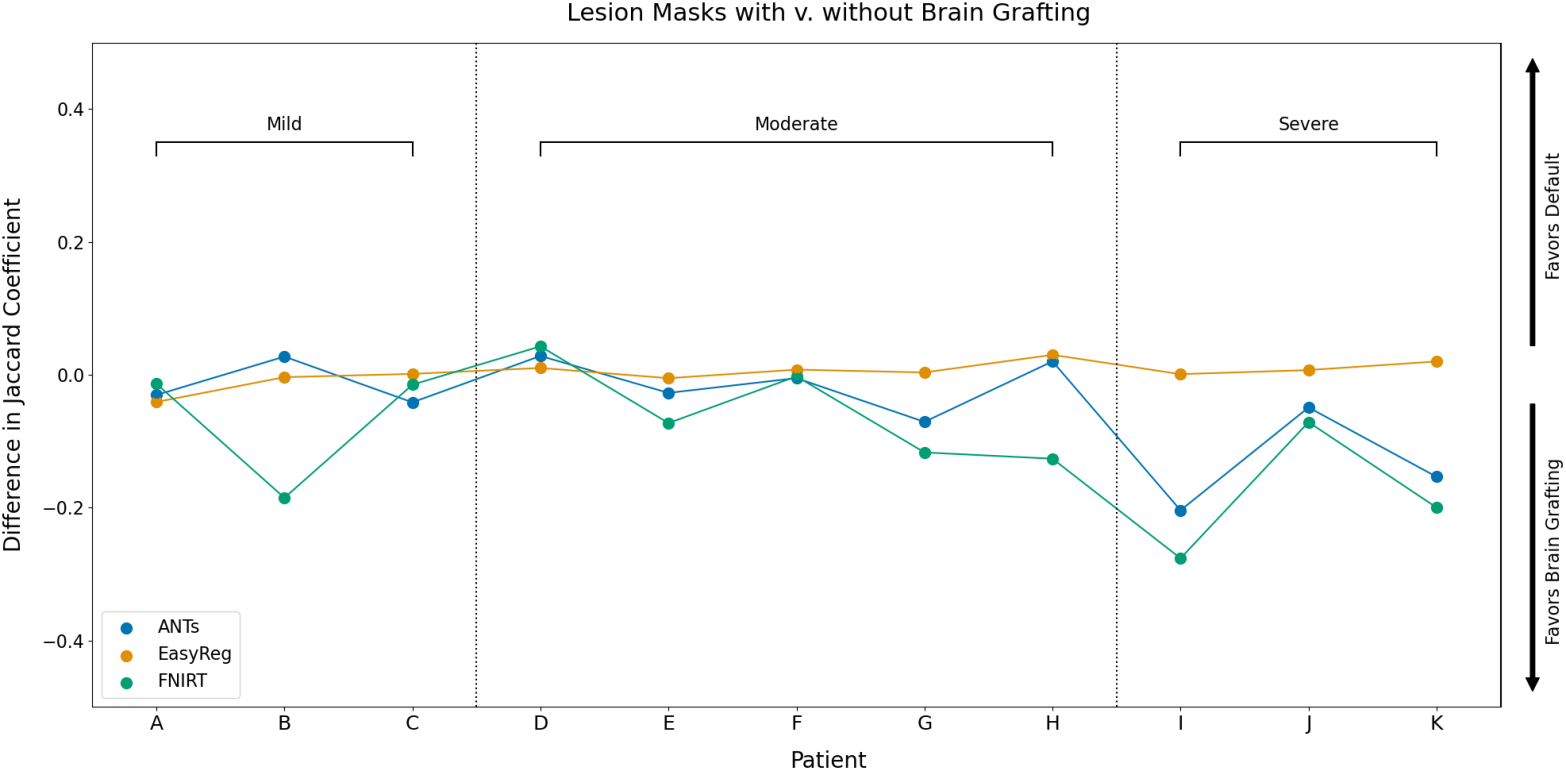
Difference in the lesion mask Jaccard coefficients from brain grafting and the default method for each patient and algorithm, grouped by lesion volume classification.

Finally, we assessed the impact of brain grafting. Paired Wilcoxon Signed-Rank Tests suggested that brain grafting had a medium positive effect on the normalization of the lesioned area for FNIRT (W = 4.00, p-adjusted = 0.0205; mean difference = -0.0725, Cohen’s d = -0.4607), while there was no significant impact for ANTs (W = 11.00, p-adjusted = 0.1611) nor EasyReg (W = 19.00, p-adjusted = 0.7207) (Fig. 7).

**Fig. 8.**
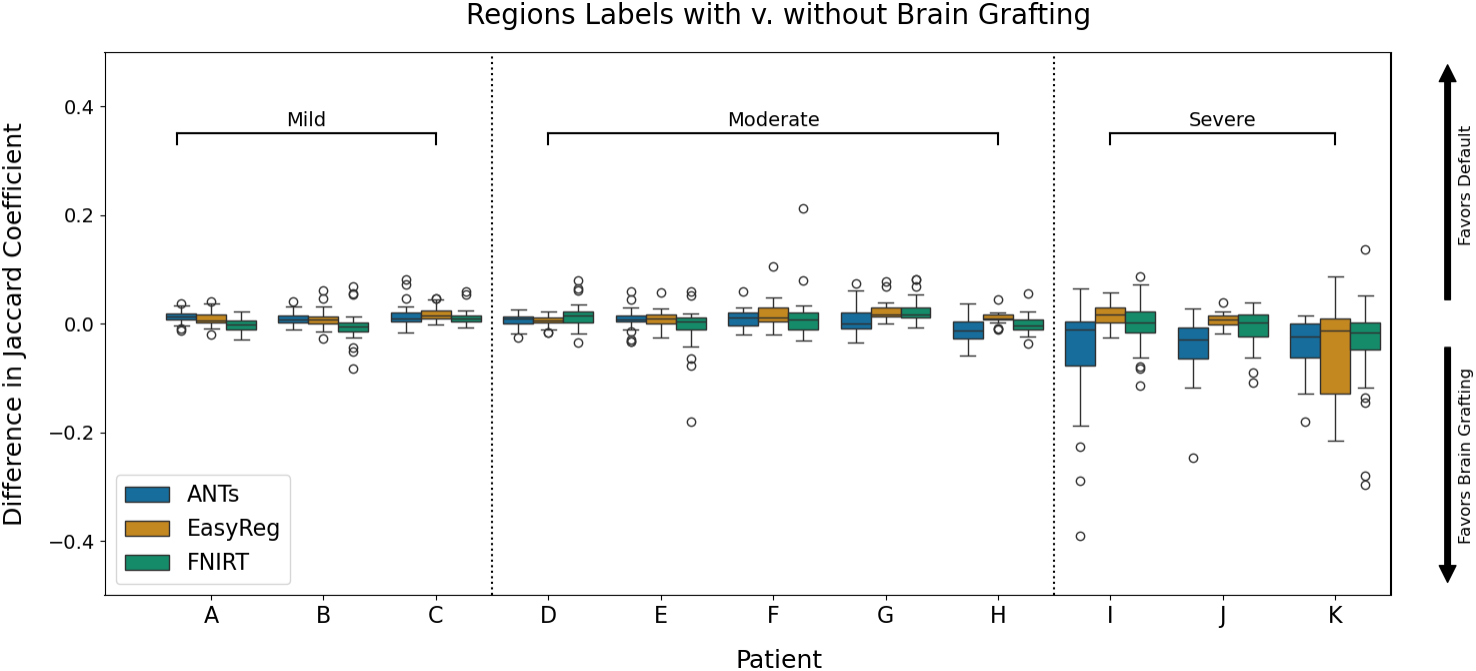
Distribution of differences in the region label Jaccard coefficients from brain grafting and the default method for each patient and algorithm, grouped by lesion volume classification.

Regarding the impact of brain grafting on the normalization of the rest of the brain, paired Wilcoxon Signed-Rank Tests suggested that brain grafting has a slight negative impact on the accuracy of EasyReg (W = 9078.00, p-adjusted <0.0001; mean difference = 0.0097, Cohen’s d = 0.0472) and FNIRT (W = 20748.00, p-adjusted = 0.0403; mean difference = 0.0037, Cohen’s d= 0.00180). There was no significant impact on the performance of ANTs (W = 24336.00, p-adjusted = 1.00) (Fig. 8).

### 3.2 Qualitative results

**Fig. 9.**
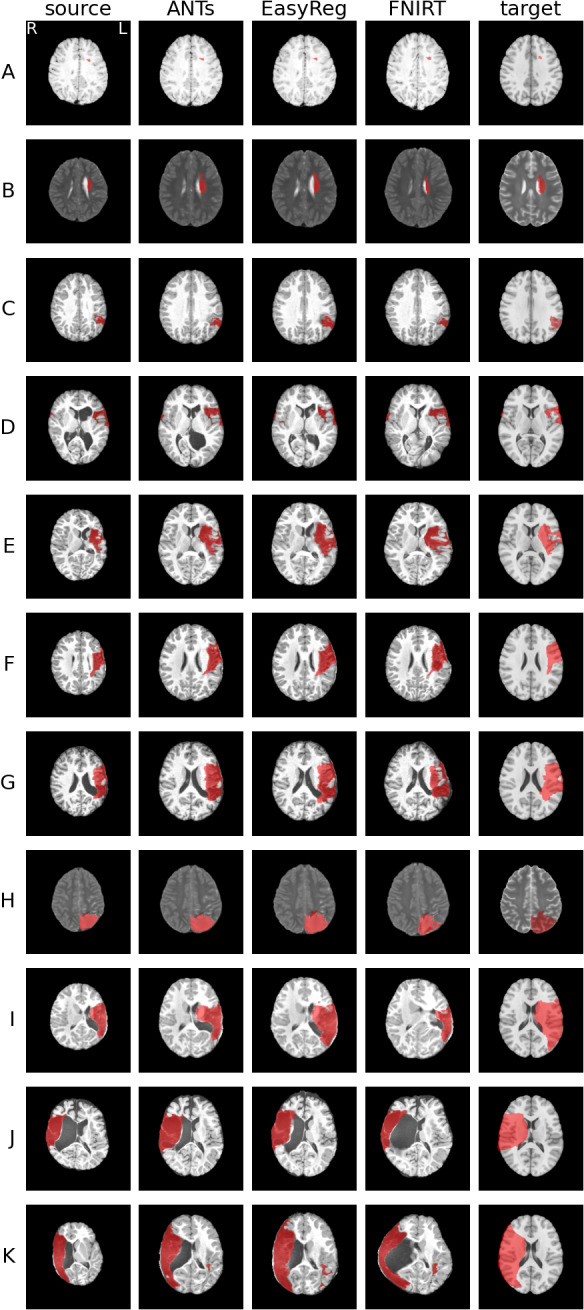
Selected slices for comparison between source brain, normalized output from each registration software, and hand-drawn target lesion mask on the target atlas for each patient.

In the blinded ranking, EasyReg was selected as the preferred software for eight of the patient brains while ANTs was preferred three times. FNIRT was ranked lowest in all 11 cases. FNIRT performed particularly worse in cases with larger lesions and often had difficulty normalizing the edge of the brain compared to the other two software packages (see Fig. 9 - patients I, K). For two of the three largest lesions, ANTs produced the preferred output, which was attributed to better normalization of enlarged ventricles than with EasyReg, although, across the cohort, neither was consistently better than the other at compensating for ventricle enlargement (see Fig. 9 - patients D, I, K).

In cases of severe hemisphere atrophy, FNIRT was able to bring the hemisphere to the correct size, but this was often accompanied by extraneous extreme local warping across the brain (see Fig. 9 - patients E, H, I, J, K), while ANTs and EasyReg generated warp fields that were more anatomically and topologically consistent. However, in less extreme cases, FNIRT was often better in correcting ventricle enlargement compared to ANTs and EasyReg, which often did not shrink the ventricles as much as needed (see Fig. 9 - patient D, E). In many cases, the performance of ANTs and EasyReg was very similar, though ANTs seemed to be more likely to reduce the extent of the lesion in a way that did not accurately reflect the amount of focal damage in the original brain.

**Fig. 10.**
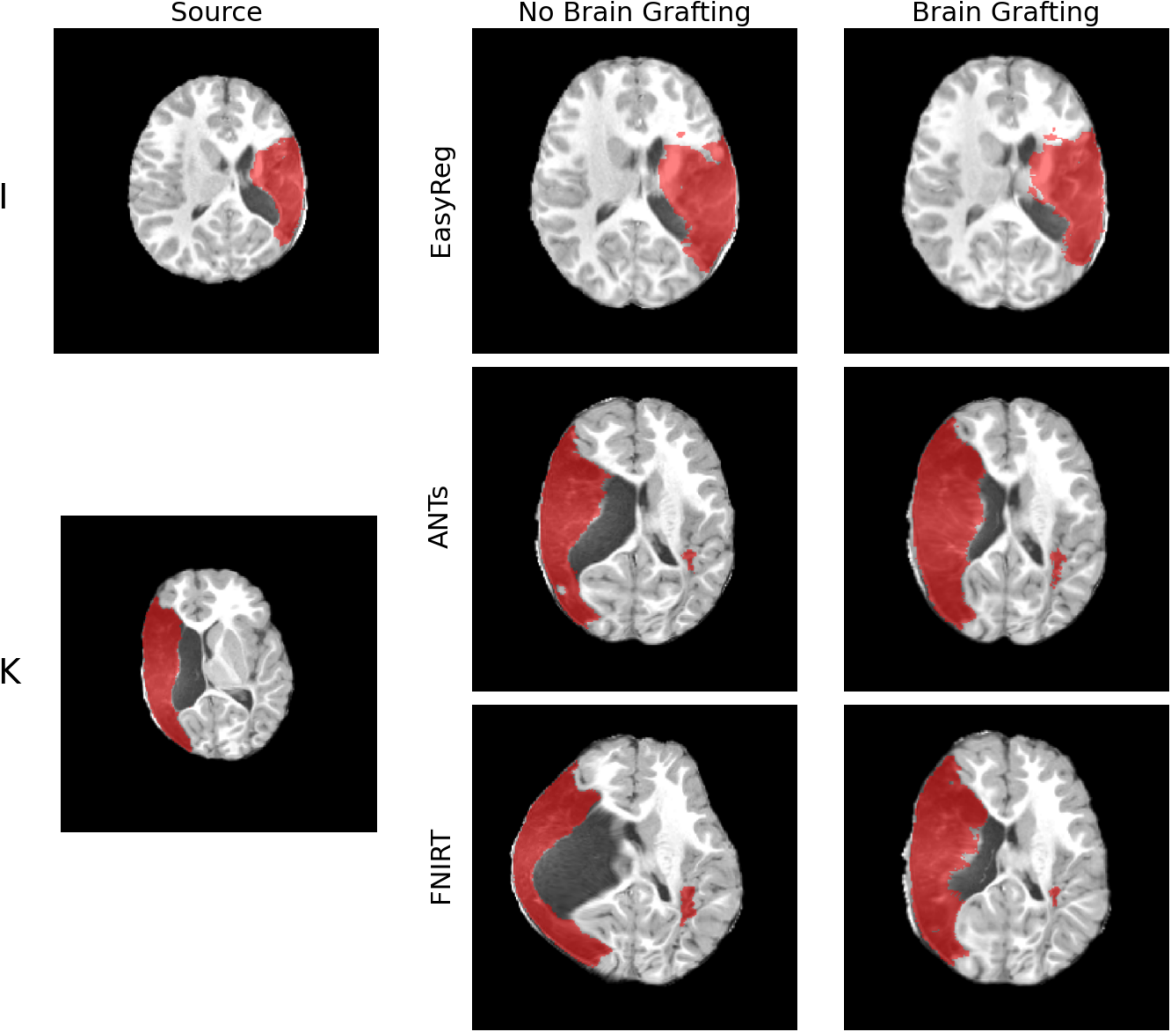
Selected brain grafting comparison examples. Patient I with EasyReg (top): brain grafting appears to reduce accuracy; note the slightly larger posterior left lateral ventricle and incorrectly reduced lesion volume with brain grafting compared to without brain grafting. Patient K with ANTs (middle): brain grafting appears to improve accuracy; note the smaller right lateral ventricle and more anatomically accurate posterior-occipital white matter with brain grafting compared to without brain grafting. Patient K with FNIRT (bottom): brain grafting appears to improve accuracy; note the smaller right lateral ventricle and more anatomically accurate brain edge.

Finally, we looked at the impact of brain grafting. In several cases, both FNIRT and ANTs seemed to gain some benefit from the addition of brain grafting; in some instances, this was very noticeable, while in other cases, the differences were relatively minor (Fig. 10). In a number of cases, brain grafting resulted in better ventricle correction and thus better focal lesion volume recapitulation (Fig. 10). There was no clear lesion presentation that seemed to predict which cases would benefit from brain grafting. Interestingly, for EasyReg, the addition of brain grafting resulted in only very minor improvements, if any at all, and in some cases seemed to cause slightly poorer performance (Fig. 10).

## 4 Discussion

Spatial normalization of brain imaging with chronic perinatal stroke involves unique challenges, due to the associated variably severe anatomical changes. The present study assesses the performance of three publicly available and commonly used software packages in the spatial normalization of chronic perinatal stroke imaging to a standard brain template. We found that EasyReg and ANTs outperformed FNIRT, based both on quantitative comparisons and visual inspection. Additionally, while the addition of brain grafting did not improve overall outcomes, it may be beneficial in specific cases. Given the reduced computational resources needed for EasyReg, as well as our apparent preference for this software based on expert visual review, we believe EasyReg is an appropriate and reasonable starting point for normalization of brains with perinatal stroke or similarly distorted anatomy.

Comprehensive comparisons of registration algorithm performance have previously been published for registration of healthy brains (Klein et al., 2009; Ou et al., 2014). This has even been compared in a lesion cohort, eight patients with brain tumors, though the use of specific lesion compensation was not included (Ou et al., 2014). It is important to understand algorithms’ ability to perform in healthy brains and in lesion cohorts without explicit compensation, given the time-consuming nature of segmentation generation. However, this differs from the goals of the current study, in which we wish to find the registration algorithm to best approximate or recapitulate the original pre-injury anatomy when lesion segmentations are available.

In our cohort, FNIRT showed reduced accuracy in the normalization of the largest lesions compared to ANTs and EasyReg. Based on visual inspection, this appears to be due to FNIRT pulling the perilesional area into the lesion territory to an extreme extent, distorting the brain edge as well as surrounding structures. Between ANTs and EasyReg, visual inspection did find a preference for EasyReg lesion normalization. This preference seems to largely stem from EasyReg’s adjustment of the lesioned area itself; EasyReg was better able to approximate the original anatomic relationship between the lesion and the surrounding tissue. Regarding the rest of the brain, the normalization performance of ANTs and EasyReg again did not statistically differ, though both ANTs and EasyReg produced normalizations which were significantly better than those resulting from FNIRT. Visual inspection suggested that FNIRT was most likely to overly deform structures in attempts to compensate for the lesioned areas.

Of note, recent work with healthy brain imaging has found that ANTs performs slightly better than EasyReg in T1w to T1w registration (Iglesias, 2023). For our perinatal stroke cohort, EasyReg may gain some benefit over ANTs due to its training data, which includes brains with strong Alzheimer-related atrophy and white matter lesions (Iglesias, 2023). Additionally, SynthSeg, the learning-based segmentation algorithm incorporated into EasyReg, has been trained on clinical-quality data (Billot, Magdamo, et al., 2023), which may also contribute to the slightly stronger performance seen here. Previous work has also reported FNIRT having trouble with the area outside the immediate neighborhood of the lesion, as well as with the edges of the brain (Ou et al., 2014). FNIRT and ANTs use similar processes to carry out registration, though there are some differences that may explain their diverging performance. While FNIRT and ANTs both use intensity-based similarity metrics, FNIRT uses sum-of-squared differences (SSD) as the default similarity metric, while ANTs uses cross-correlation (CC). Additionally, the cost-function-masking (CFM) used by the two software packages is slightly different, as ANTs uses a modified version called constrained cost-function-masking, which estimates the deformation field for the lesioned area rather than leaving it unspecified, which is particularly helpful in the case of large or bilateral lesions (Kim et al., 2007). It is important to note that while FNIRT did not perform well in our specific study cohort, it is still useful for healthy brain imaging and has the benefit of being less computationally intensive than ANTs. Additionally, FNIRT has many parameters that knowledgeable users can tune to enhance its performance.

While differences in CFM may have factored into the variation between FNIRT and ANTs, overall, in our cohort, CFM was often not sufficient to ensure accurate spatial normalization. Previous work looked at CFM in real chronic stroke scans and found it to be beneficial, especially in cases of secondary effects from lesions (Andersen et al., 2010), and there have been many papers with similar findings in healthy brains with simulated lesions (Brett et al., 2001; Hanayik, 2018; Ripollés et al., 2012). Thus, CFM is certainly beneficial, but it does not account for differences that occur outside the lesioned area, such as secondary chronic white matter changes or enlarged ventricles. Of note, much of the literature focusing on this topic leverages simulated lesions, which allow for the generation of an actual “ground truth” deformation field for comparison, but often do not capture the perilesional intensity differences, regional deformations, and remote effect lesions often have on the rest of the brain. Interestingly, while brain grafting is based on the same principle as CFM (both remove the lesioned tissue from the registration computation), within our cohort, normalization with brain grafting did demonstrate some impacts over normalization with CFM in some cases. In particular, FNIRT with brain grafting showed statistical and visual improvements in the normalization of the lesion masks, lending support to the idea that the poor performance with larger lesions may be due in part to compensation for large areas of exclusion from the cost function calculations.

Overall, we find notable differences in the software packages and factors that impact their performance in normalizing chronic stroke imaging. Ultimately, the goal is to align preserved structural anatomy to a sufficient level to accurately capture the area of focal injury in a common frame of reference to allow for between-patient comparisons. Lesion-focused methods such as VLSM/LNM present a particular difficulty, because they require accurate normalization of the injured hemisphere to pre-injury levels. Normalization algorithms are often constructed to be optimal for typical brains that are already similar to the template, and a penalty is applied for high deformation. Compensation techniques, such as cost function masking, can help by allowing the algorithm to bias the transform to tissue outside of the lesioned area. These techniques and algorithms are successful in cases of adult stroke and other lesion types, as discussed in the introduction, likely due to the relatively intact structure of the brain. However, we have found that in chronic perinatal stroke, the downstream effects outside of the focal-injury area are not well-accounted for and distort the overall registration. Since perinatal strokes occur very early in development, these secondary impacts are often more pronounced. Distorted anatomy, such as enlarged ventricles, are not able to be fully warped back into place likely due to limitations placed on the algorithm parameters and the dissimilarity to the template. In cases of larger lesions and/or high anatomical deformation, this can be even more of a challenge. For other use cases, like longitudinal within-patient registration, where the similarity to the target is higher, or research focusing on preserved tissue, this may be less of an issue.

From a practical standpoint, as has been alluded to throughout the paper, for certain datasets, a current necessitated practice for creating lesion masks is to manually draw the lesion onto a template by essentially doing a “mental warp” of the brain. This is the procedure we used to create our “target” lesion labels for comparison. This is a difficult task and has issues of reliability and reproducibility. Other potentially more-efficient options include warping to a template then applying manual corrections or repeating linear warps recursively (Mandal et al., 2020), however the fidelity of these methods is usually similar to direct tracing In fact, published protocols demonstrate that, with training and quality control, manual segmentation achieves high inter- and intra-rater reliability and thus continues to serve as ground truth in pediatric and adult cohorts (Cariola et al., 2025; Everts et al., 2023; Lo et al., 2022; Vossough et al., 2024). In the present study, the sample size was limited to allow for more careful hand-drawing of all lesions in template space to maintain a consistent benchmark for comparison across the degree of distortion. Additionally, we conducted analyses of the non-lesioned areas and performed a qualitative assessment to help account for limitations in the available ‘ground truth’. Ultimately, accurate spatial normalization of lesioned areas would remove the necessity of all of these workarounds, increasing the accuracy and reliability of the template space lesion masks.

It’s important to note that EasyReg is still a newly available software, and a version specifically trained on lesioned data is feasible and would be of great benefit. Furthermore, ANTs and FNIRT have many parameters that could be optimized to enhance their performance; tuning these for specific cases requires a high degree of familiarity and expertise with either software package. Future work could assess the accuracy of different parameters or other ways to compensate for the default deformation limitations set by the algorithms that don’t require expert parameter tuning. Additionally, future research with larger sample sizes and different lesion types could help identify brain and lesion features that predict the success of various algorithms and compensation techniques.

This study has a few notable limitations: first, we compared the results of spatially normalizing 11 brains; a larger cohort would both improve statistical power and allow us to assess whether other patient variables, such as age at the time of imaging, impact performance. Second, we did not compare the performance of FNIRT and ANTs without generating a lesion mask and employing CFM; prior studies have already demonstrated that masking produces better results (Andersen et al., 2010; Brett et al., 2001; Hanayik, 2018; Ripollés et al., 2012). Third, and most notably, we do not have a ground truth to use when determining the “best” normalization. Here, we use lesion masks hand-drawn in template space, with a specific focus on localizing the initial injury territory, as such projects focusing on other injury effects, such as secondary white matter tract loss or post-injury connectivity changes, may have a different optimal normalization. Nonetheless, our method tests registration software with real-world lesions, across a range of distorted anatomy without resorting to synthetic or simulated lesions.

In conclusion, our results suggest that for brains with chronic focal lesions EasyReg or ANTs are both reasonable options for spatial normalization. If outputs are very poor, using brain grafting with ANTs may improve results in some cases. However, when accurate pre-injury estimation of lesion segmentations in a standard atlas space are needed, it may still be necessary to hand correct outputs, especially in cases where the extra-lesional deformations are significant. We believe our findings may have useful implications for other lesion types and structural anomalies as well, including tumors and surgical resections, as well as normal anatomical variants such as enlarged ventricles which can similarly cause difficulty for normalization algorithms. We remain optimistic about the feasibility of fully automated approaches to lesion segmentation and normalization for anatomically variable clinical data but recommend caution in utilizing these software packages without consistent expert inspection and quality control protocols for now.

## Supplementary Material

Supplementary Material

## Data Availability

Patient imaging data and lesion segmentations are available contingent upon a Data Use Agreement (DUA) approved by Massachusetts General Hospital, in compliance with institutional and regulatory requirements. Software is publicly available (FNIRT: Hoffmann et al., 2022; Iglesias, 2023); EasyReg: Billot, Magdamo, et al., 2023); ANTs: https://stnava.github.io/ANTs/). Example scripts are available from our publicly available github (https://github.com/bchcohenlab/Perinatal_Stroke_Brain_Normalization).
