# Supplementary material for: Comparison of brain normalization software and lesion compensation techniques in chronic perinatal stroke imaging
Source: Imaging Neurosci (Camb). 2025 Dec 3;3:IMAG.a.1048. doi: 10.1162/IMAG.a.1048 (PMC13288496; doi:10.1162/IMAG.a.1048)
Supplement: Supplementary Material [file IMAG.a.1048_supp.pdf]

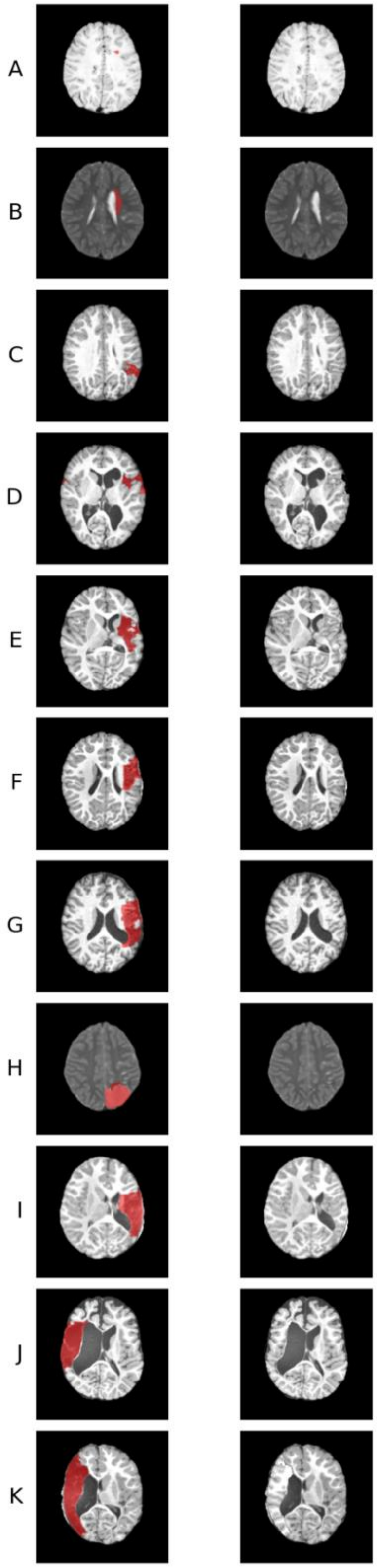

Supplemental Materials Figure 1 - Selected slices of brains following the brain grafting procedure with (left) and without (right) the lesion area denoted for each patient.
